# The causal effect of family physician program on the prevalence, screening, awareness, treatment, and control of hypertension and diabetes mellitus in an Eastern Mediterranean Region: a causal difference-in-differences analysis

**DOI:** 10.1186/s12889-023-16074-z

**Published:** 2023-06-15

**Authors:** Neda Mohammadi, Ahad Alizadeh, Sahar Saeedi Moghaddam, Erfan Ghasemi, Naser Ahmadi, Mehdi Yaseri, Negar Rezaei, Mohammad Ali Mansournia

**Affiliations:** 1 Department of Epidemiology and Biostatistics, School of Public Health, Tehran University of Medical Sciences, Tehran, Iran; 2 Medical Microbiology Research Center, Qazvin University of Medical Sciences, Qazvin, Iran; 3 Endocrinology and Metabolism Population Sciences Institute, Non-Communicable Diseases Research Center, Tehran University of Medical Sciences, Tehran, Iran; 4 Endocrinology and Metabolism Research Center, Endocrinology and Metabolism Clinical Sciences Institute, Tehran University of Medical Sciences, Tehran, Iran; 5 Kiel Institute for the World Economy, Kiel, Germany

**Keywords:** Family physician program, Hypertension, Diabetes mellitus, Difference-in-difference, TMLE, Iran

## Abstract

**Background:**

Hypertension (HTN) and diabetes mellitus (DM) as part of non-communicable diseases are among the most common causes of death worldwide, especially in the WHO’s Eastern Mediterranean Region (EMR). The family physician program (FPP) proposed by WHO is a health strategy to provide primary health care and improve the community’s awareness of non-communicable diseases. Since there was no clear focus on the causal effect of FPP on the prevalence, screening, and awareness of HTN and DM, the primary objective of this study is to determine the causal effect of FPP on these factors in Iran, which is an EMR country.

**Methods:**

We conducted a repeated cross-sectional design based on two independent surveys of 42,776 adult participants in 2011 and 2016, of which 2301 individuals were selected from two regions where the family physician program was implemented (FPP) and where it wasn't (non-FPP). We used an Inverse Probability Weighting difference-in-differences and Targeted Maximum Likelihood Estimation analysis to estimate the average treatment effects on treated (ATT) using R version 4.1.1.

**Results:**

The FPP implementation increased the screening (ATT = 36%, 95% CI: (27%, 45%), *P*-value < 0.001) and the control of hypertension (ATT = 26%, 95% CI: (1%, 52%), *P*-value = 0.03) based on 2017 ACC/AHA guidelines that these results were in keeping with JNC7. There was no causal effect in other indexes, such as prevalence, awareness, and treatment.

The DM screening (ATT = 20%, 95% CI: (6%, 34%), *P*-value = 0.004) and awareness (ATT = 14%, 95% CI: (1%, 27%), *P*-value = 0.042) were significantly increased among FPP administered region. However, the treatment of HTN decreased (ATT = -32%, 95% CI: (-59%, -5%), *P*-value = 0.012).

**Conclusion:**

This study has identified some limitations related to the FPP in managing HTN and DM, and presented solutions to solve them in two general categories. Thus, we recommend that the FPP be revised before the generalization of the program to other parts of Iran.

## Introduction

Hypertension (HTN) and diabetes mellitus (DM) as non-communicable diseases (NCD) are among the most common causes of death worldwide [1–3]. The prevalence of HTN is rising globally, and in 2015, 1.13 billion of the world’s adults had hypertension which was predicted to increase to 1.56 billion in 2025 [4–6]. Furthermore, the prevalence of DM is also growing globally. In 2017, it was estimated that 425 million people had DM [7], which was expected to increase to 629 million in 2045 [8].

The Eastern Mediterranean region (EMR) consists of 22 countries, with over 72% of them classified as low and middle-income countries. The changes in HTN prevalence are not uniform across all countries; high-income countries experienced a slight decrease, while in LMICs, the prevalence had increased [9]. In 2019, 82% of all people with HTN lived in low and middle-income countries [1].

Iran, situated in Western Asia and being one of the Eastern Mediterranean Region (EMR) countries, is experiencing an increase in the prevalence of non-communicable diseases. According to the Statistical Center of Iran, the country had a population of 75.15 million in 2011 and 79.92 million in 2016. Additionally, the estimated population sizes for 2021 and 2026 are 84.1 million and 87.7 million, respectively [10]. Based on the meta-analysis conducted in 2019, the overall HTN prevalence in Iran was 25% [11], and the overall prevalence of pre-diabetes and diabetes was 12% and 10.2%, respectively [12].

Diagnostic methods of HTN and DM are convenient by measuring blood pressure (BP) and fast blood sugar (FBS). Studies have shown that the awareness, treatment, and control of HTN and DM increased in high-income countries. However, the slope of increase in awareness and treatment of HTN and DM in low- and middle-income countries was less than in high-income countries [9, 12–14]. According to the World Health Organization (WHO) prediction, the number of deaths from non-communicable diseases will rise to 55 million by 2030 under a business-as-usual scenario in which no supplementary policy administration occurs.

The family physician program (FPP), defined by the World Health Organization (WHO), is one policy aimed at managing non-communicable diseases (NCDs). The goals of the FPP include providing primary healthcare services, improving quality and equity, and reducing costs associated with healthcare systems[15]. To provide primary health care, family physicians can play an essential role in diagnosing, controlling, and treating HTN and DM as non-communicable diseases. In the 1960s, the FPP was established in Canada and Britain, and in 1969 it was differently set up in the United States as a specialty requiring postgraduate education. Currently, the FPP is expanding to other parts of the world, including some countries in the Eastern Mediterranean region. In Iran, one of the EMR countries, the FPP was launched in June 2005 in rural areas [16]. In 2012, the FPP was implemented as a pilot study in the cities of Fars and Mazandaran provinces in Iran; nevertheless, it has not yet been expanded throughout the country [15, 17]. Despite insufficient evidence of the FPP's efficiency based on the pilot study, Iran's government decided to expand the program to all provinces in 2022 and 2023. While the specific factors that influenced this decision remain unclear, it is plausible that reduced health inequalities, decreased health costs, and a desire to enhance public health outcomes were contributing factors. Regardless of the rationale behind the decision, it is imperative to closely monitor the implementation of the expanded FPP and continue to evaluate its impact in Iran.

One of the FPP's goals is to improve the community awareness of HTN and DM, which is crucial to reduce the delay of treatment initiation and preventing related mortality. However, previously published reports declared that FPP has challenges in implementation in Iran and needs to be improved in several aspects [16, 18, 19].

Based on our knowledge, no study investigated the causal effect of the FPP on HTN and DM in Iran. The primary aim of this study is to investigate the causal effect of the FPP implementation on the prevalence, screening, awareness, treatment, and control of HTN and DM, two important type of NCDs, in Iran using an Inverse-Probability Weighting Difference-In-Differences (IPW Diff-in-Diff) analysis. We also used targeted maximum likelihood estimation (TMLE) as a secondary analysis to examine the causal impact of the Family Physician Program (FPP) implementation on systolic blood pressure (SBP), diastolic blood pressure (DBP), and fasting blood sugar (FBS) levels.

## Materials and methods

### Study design

We conducted a repeated cross-sectional design based on two population-based surveys from non-communicable diseases risk factors survey in Iran, known as STEPS. This survey is an ongoing sequential cross-sectional population-based study based on an approach designed by the WHO. This survey aims to monitor NCD risk factors on a national level. A total of eight STEPS cross-sections have been conducted in Iran (2005, 2006, 2007, 2008, 2009, 2011, 2016, 2021). For each cross-section, a proportional-to-size sampling using a cluster random sampling frame was employed to independently select samples from each province's rural and urban areas across all 31 provinces of Iran. The minimum sample size was determined with a 95% confidence level, using the province with the lowest population density as the basis for calculations. Other provinces' sample sizes were based on their population ratios to this province. To account for non-response error and sampling design, an additional 10% was added to the estimated sample size [20].

According to this study’s aim and diff-in-diff concepts, we selected two cross-sections of the STEPS survey The first survey was conducted in 2011, one year before the implementation of the FPP (pre-policy), and the second survey was conducted in 2016, four years after the FPP was implemented (post-policy). To compare interest indicators before and after policy implementation, and estimate the average treatment effect on the treated city (ATT), two cities were selected for the analysis. The first city selected was Shiraz, which was designated as the FPP (treated) city, while the second city, Mashhad, was chosen as the non-FPP (control).

### Sources of data

This study utilized data from two independent cross-sections of the STEPS survey conducted in 2011 and 2016. All individuals aged 18 years and above who were interested in participating in the STEPS survey were eligible for inclusion in this study. Pregnant women were excluded from our analysis, and participants under the age of 25 were excluded from our diabetes analysis. In total, 2301 participants aged 18 years or above were selected from the two cross-sections, with 658 adults in 2011 and 1643 adults in 2016. Approximately 41% of the samples were in the treated group and 59% were in the control group.

### Variables

The primary interest outcomes were prevalence, ever screening, awareness, treatment, and control of HTN and DM to evaluate the performance of the some mentioned aims of FPP. Systolic blood pressure (SBP), diastolic blood pressure (DBP), and FBS were chosen as secondary outcomes.

Based on JNC7, high blood pressure was defined as SBP ≥ 140 mmHg or DBP ≥ 90 mmHg [21]; whereas, based on 2017 ACC/AHA, SBP ≥ 130 mmHg or DBP ≥ 80 mmHg was considered high blood pressure [22]. In this study, people with the following characteristics were classified as hypertensive:a) High blood pressure definition according to the JNC7 and 2017 ACC/AHA guidelines separately, b) the self-reported use of antihypertensive drugs in the last two weeks, c) self-reported previous diagnosis of hypertension by the physician.

Furthermore, diabetes is defined as:a) having fasting blood sugar (FBS) levels ≥ 126 mg/dL, b) the self-reported use of antidiabetic drugs in the last two weeks, c) self-reported previous diagnosis of diabetes by the physician.

Ever-screening of HTN (or DM) was considered as if a hypertensive (or diabetes) answered ‘Yes’ to the question ‘Have you ever been measured blood pressure (or fast blood glucose) by a physician or a health professional? Awareness of HTN (or DM) was considered as if screened hypertensive (or diabetes) patients answered ‘Yes’ to the question ‘Have you ever been diagnosed with hypertension (or diabetes) by a physician or a health professional? Treatment was defined as the self-reported taking of antihypertensive treatment (or antidiabetic treatment) among aware individuals. Hypertension control referred to an average SBP < 140 and DBP < 90 mmHg based on the JNC7 and an average SBP < 130 and DBP < 80 mmHg based on the 2017 ACC/AHA among participants who were on antihypertensive treatment. Diabetes control referred to an average FBS < 126 mg/dL among participants who were taking treatment.

### Parallel trend adjustment

The diff-in-diff analysis relies on an important assumption called the parallel trend assumption (PTA) to accurately estimate the Average Treatment Effect on the Treated (ATT). This study evaluated the following covariates to hold the PTA assumption in the IPW diff-in-diff analysis: age, body mass index (BMI), waist-to-hip ratio (WHR), triglyceride, FBS, gender (female and male), education (no education, 1–6 years, 7–12 years, and more than 12 years education), job status (employee, unemployment, housekeeper, retired), current smoking (never smoker/non-smoker, current daily cigarette smoker), past smoking (never smoker, smoker), alcohol consumption (alcohol drinkers vs. non-drinkers), consumption of fruit, vegetable, fish and fast food (number of days per a week), consumption of dairy (times per a day), table salt usage, dyslipidemia (referred to either total cholesterol ≥ 200 mg/dL, high-density lipoprotein, cholesterol < 35 mg/dL, or low-density lipoprotein cholesterol ≥ 130 mg/dL), physical activity (sufficient, insufficient), and wealth status.

Insufficient physical activity is defined as physical activity less than 600 metabolic equivalent tasks (MET) minutes per week as proposed by WHO [23]. The wealth status was measured by the wealth index [24] using Principal Component Analysis, and it was grouped into five equally subsets from poorest to richest (poorest, poorer, middle, richer, or richest) based on the quintiles of principal component scores.

### Statistical methods

One of the most commonly used approaches for evaluating new policies is the difference-in-differences (Diff-in-Diff) method, particularly when conducting randomized trial studies is not feasible [25, 26]. The causal diff-in-diff method is interested in comparing outcomes of treated individuals under both treatment (Y_t_^1^) and non-treatment (Y_t_^0^) conditions (counterfactual outcomes). These models take the form:

$$E\left({Y}_{t}^{1}-{Y}_{t}^{0 }| A=1\right)$$

where A = 1 indicate the treated group.

Note that *Y*^1^ and *Y*^0^ are potential outcomes in the sense that we only observe one of them for each person. In ATT estimand, we only observed the *Y*^1^ in treated group. Thus, in diff-in-diff approach, to impute the untreated outcomes in the treated group and determine temporal variation in the outcomes that are not due to exposure, a control group (non-FPP) is defined. This group helps to obtain an appropriate counterfactual estimation of the causal effect.

However, the diff-in-diff method relies on strong assumptions called the parallel trend (PT) assumption. The parallel trend assumption states that average outcomes for the treated and control groups would have followed parallel paths over time in the absence of treatment.

This study employed the inverse probability weighting difference-in-differences analysis developed by Abadie (2005) to estimate the effect of treatment (physician family program) on the outcome variables in the repeated cross-sectional data. One of the advantages of Abadie's work is that it can relax the PT assumption by assuming that after conditioning on covariates, PT assumption holds. IPW method allows for constructing a counterfactual response and reduces selection bias. Abadie assumes that the pooled repeated cross-section data are independent and identically distributed, drawing from the mixture distribution. IPW-approach avoids directly modeling the outcome. In this approach, when repeated cross-section data are available, ATT can be formulated as:

$$ATT=\frac{1}{E\left(A\right)}E\left[\frac{A-\widehat{P}\left(A=1| X\right) T-\uplambda }{1-\widehat{P}\left(A=1| X\right)\uplambda \left(1-\uplambda \right)}Y\right]$$

where* λ* $\upepsilon$ *(*0*,* 1*)* reflects the proportion of the observations sampled in the post-treatment period.

Furthermore, we employed the Targeted Maximum Likelihood Estimation (TMLE) to investigate the effect of FPP on systolic, diastolic, and FBS measures. TMLE is a two-step method to construct the estimators for the parameter of interest, allowing machine learning algorithms to minimize the risk of model misspecification. In the first step, it obtains an estimate of the data-generating distribution using the Super-Learner (SL) algorithm. Then, the second step updates the initial fit through a fluctuation step targeted toward the parameter of interest. The TMLE method as formulated:

$$\mathrm{logit}\left(\mathrm{E}\left(\mathrm{Y}|\mathrm{A},\mathrm{W}\right)\right)=\mathrm{logit}\left(\mathrm{E}\left(\mathrm{Y}|\mathrm{A},\mathrm{W}\right)\right)+\varepsilon H\left(A,W\right)$$

where A indicates the treatment, and $\varepsilon$ is a fluctuation parameter. The parameter was estimated using maximum likelihood by setting the initial fit as an offset in the model, and H(A,w) is a function of the propensity score that is determined by the influence function concept.

TMLE is efficient and doubly robust, which means that if either the outcome model or treatment model is incorrectly specified, the estimate of TMLE is consistent.

The descriptive results are reported using mean ± standard deviation, n (%), or median (interquartile range: IQR). Fisher's exact test was used to compare the distribution of binary qualitative variables between two groups. The mean or median of quantitative variables between two groups was evaluated using the t-test or Mann–Whitney U test, respectively. All confidence intervals (CI) were estimated based on 95% confidence, and the *P*-value less than 0.05 was considered significant. All analyses were done separately for diabetes and hypertension using the software program R version 4.1.1.

## Results

The non-response rates for the first and second surveys were approximately 3% and 2%, respectively. Characteristics of 2301 respondent participants in the FPP and non-FPP cities in the pre-policy (2011) and post-policy (2016) periods are summarized in Table 1. Among the 658 individuals included in our pre-policy data, 336 (51.06%) were residents in the FPP-administered city. In post-policy, 1643 individuals were included in the study, of which 609 (37.07%) were defined as residents in the FPP city. The mean ± SD age of participants in the pre-policy was 41.93 ± 15.64, and 58% were female. In post-policy, the mean ± SD age was 45.10 ± 16.00, and 54% were female. Based on the pre and post-policy datasets, other demographic and descriptive characteristics of the FPP and non-FPP were compared and presented in Table 1.

**Table 1:** Comparing demographic and descriptive characteristics of the FPP and non-FPP based on the 2011 and 2016 surveys

Variables	Levels	Pre-policy (2011)				Post-policy (2016)			
		Total (*n* = 658)	non-FPP (*n* = 322)	FPP (*n* = 336)	*P*-value	Total (*n* = 1643)	non-FPP (*n* = 1034)	FPP (*n* = 609)	*P*-value
Age (years)		41.93 ± 15.64	42.22 ± 15.30	41.66 ± 15.99	0.648	45.10 ± 16.00	44.20 ± 15.83	46.62 ± 16.17	0.003
BMI (kg/m^2^)		26.12 ± 5.18	26.62 ± 5.55	25.63 ± 4.76	0.015	26.80 ± 4.82	26.68 ± 4.73	27.00 ± 4.97	0.213
WHR		0.90 ± 0.12	0.90 ± 0.13	0.90 ± 0.11	0.571	0.89 ± 0.09	0.88 ± 0.09	0.89 ± 0.09	0.011
Gender	Male	279 (42.4%)	143 (44.41%)	136 (40.48%)	0.349	752 (45.77%)	497 (48.07%)	255 (41.87%)	0.015
Education	no schooling	87 (13.22%)	39 (12.11%)	48 (14.29%)	0.442	107 (6.51%)	66 (6.38%)	41 (6.73%)	0.905
	1–6 years	108 (16.41%)	49 (15.22%)	59 (17.56%)		343 (20.88%)	221 (21.37%)	122 (20.03%)	
	7–12 years	317 (48.18%)	155 (48.14%)	162 (48.21%)		767 (46.68%)	483 (46.71%)	284 (46.63%)	
	> > 12	146 (22.19%)	79 (24.53%)	67 (19.94%)		426 (25.93%)	264 (25.53%)	162 (26.6%)	
Job	Employee	159 (25.48%)	75 (24.59%)	84 (26.33%)	0.024	578 (36.72%)	390 (39.08%)	188 (32.64%)	0.028
	Unemployment	109 (17.47%)	44 (14.43%)	65 (20.38%)		135 (8.58%)	81 (8.12%)	54 (9.38%)	
	Housekeeper	293 (46.96%)	146 (47.87%)	147 (46.08%)		692 (43.96%)	433 (43.39%)	259 (44.97%)	
	Retired	63 (10.1%)	40 (13.11%)	23 (7.21%)		169 (10.74%)	94 (9.42%)	75 (13.02%)	
Current smoking		54 (8.21%)	24 (7.45%)	30 (8.93%)	0.581	103 (6.27%)	61 (5.9%)	42 (6.9%)	0.449
Past smoking		23 (3.77%)	14 (4.68%)	9 (2.89%)	0.305	157 (9.76%)	91 (8.97%)	66 (11.11%)	0.172
Alcohol use		38 (5.86%)	16 (5.02%)	22 (6.67%)	0.408	154 (9.61%)	73 (7.22%)	81 (13.71%)	0
Dairy Consumption (times per a day)	< 1	239 (37.52%)	117 (36.79%)	122 (38.24%)	0.445	590 (36.71%)	434 (42.8%)	156 (26.31%)	0
	1	255 (40.03%)	123 (38.68%)	132 (41.38%)		700 (43.56%)	431 (42.5%)	269 (45.36%)	
	≥ 2	143 (22.45%)	78 (24.53%)	65 (20.38%)		317 (19.73%)	149 (14.69%)	168 (28.33%)	
Fruit Consumption (days per a week)		5.00 (3.00, 7.00)	5.00 (3.00, 7.00)	5.00 (3.00, 7.00)	0.628	4.00 (3.00, 7.00)	4.00 (3.00, 7.00)	5.00 (3.00, 7.00)	0.2
	≥ 1	639 (97.11%)	318 (98.76%)	321 (95.54%)	0.02	1560 (94.95%)	984 (95.16%)	576 (94.58%)	0.63
Vegetable Consumption (days per a week)		4.00 (2.00, 7.00)	4.00 (2.00, 7.00)	5.00 (3.00, 7.00)	0.149	5.00 (3.00, 7.00)	5.00 (3.00, 7.00)	5.00 (3.00, 6.00)	0.13
	≥ 1	631 (95.9%)	310 (96.27%)	321 (95.54%)	0.714	1555 (94.64%)	988 (95.55%)	567 (93.1%)	0.048
Fast food Consumption (times per a week)	0	455 (70.32%)	241 (76.51%)	214 (64.46%)	0.001	1344 (83.48%)	876 (86.22%)	468 (78.79%)	0
	1	105 (16.23%)	46 (14.6%)	59 (17.77%)		192 (11.93%)	100 (9.84%)	92 (15.49%)	
	≥ 2	87 (13.45%)	28 (8.89%)	59 (17.77%)		74 (4.6%)	40 (3.94%)	34 (5.72%)	
Fish Consumption (times per a week)	0	365 (57.94%)	189 (61.17%)	176 (54.83%)	0.231	1099 (68.26%)	788 (77.56%)	311 (52.36%)	0
	1	179 (28.41%)	83 (26.86%)	96 (29.91%)		396 (24.6%)	198 (19.49%)	198 (33.33%)	
	≥ 2	86 (13.65%)	37 (11.97%)	49 (15.26%)		115 (7.14%)	30 (2.95%)	85 (14.31%)	
Table salt		367 (56.03%)	198 (62.07%)	169 (50.3%)	0.005	712 (44.2%)	558 (54.87%)	154 (25.93%)	0
Physical activity	Sufficient	179 (43.87%)	168 (56.19%)	11 (10.09%)	0	591 (39.22%)	369 (39.26%)	222 (39.15%)	1

The results are reported by mean ± SD, n (%), or median (first quantile, third quantile)

### Hypertension

The results of the IPW Diff-in-Diff analysis to determine the effect of FPP on HTN prevalence, screening, awareness, treatment, and control are demonstrated in Table 2. Among hypertensives, based on 2017 ACC/AHA guidelines, the results showed that FPP led to a significant increase in HTN screening (ATT = 36%, 95% CI: (27%, 45%)) and control (ATT = 26% (1%, 52%)), compared to the non-FPP group. There was no causal effect in other indicators, such as prevalence, awareness, and treatment.

**Table 2:** Effect of FPP on the prevalence, ever screening, awareness, treatment, and controlling of hypertension in the pre and post-policy periods based on 2017 ACC/AHA

Outcome	Pre-policy (2011)			Post-policy (2016)			ATT (95% CI)	*P*-value
	non-FPP (*n* = 322)	FPP (*n* = 336)	*P*-value	non-FPP (*n* = 1034)	FPP (*n* = 609)	*P*-value		
Prevalence	169 (52.48)	178 (52.98)	0.938	517 (50)	270 (44.33)	0.028	-7 (-16, 2)	0.109
Ever screening	158 (93.49)	113 (63.84)	< 0.001	432 (84.21)	246 (91.11)	0.008	36 (27, 45)	< 0.001
Awareness	63 (39.87)	55 (48.67)	0.172	199 (46.06)	136 (55.28)	0.025	1 (-14, 16)	0.856
Treatment	43 (69.35)	39 (70.91)	1	131 (67.18)	80 (59.26)	0.162	-10 (-30, 10)	0.342
Controlling	10 (23.26)	8 (21.05)	1	23 (17.56)	29 (36.25)	0.003	26 (1, 52)	0.030

The results based on JNC7 were in keeping with ACC/AHA guidelines, and they showed that FPP implementation had an increased causal effect on the HTN screening (ATT = 22%, 95% CI: (12%, 32%)) and the control (ATT = 26%, 95% CI: (1%, 51%)), compared with non-FPP (Table 3).

**Table 3:** Effect of FPP on the prevalence, ever screening, awareness, treatment, and controlling of hypertension in the pre and post-policy periods based on JNC 7

Outcome	Pre-policy (2011)			Post-policy (2016)			ATT (95% CI)	*P*-value
	non-FPP (*n* = 322)	FPP (*n* = 336)	*P*-value	non-FPP (*n* = 1034)	FPP (*n* = 609)	*P*-value		
Prevalence	94 (29.19)	89 (26.49)	0.486	303 (29.3)	175 (28.74)	0.822	2 (-7, 10)	0.669
Ever screening	91 (96.81)	69 (77.53)	< 0.001	282 (93.07)	167 (95.43)	0.328	22 (12, 32)	< 0.001
Awareness	63 (69.23)	55 (79.71)	0.15	199 (70.57)	136 (81.44)	0.013	1 (-15, 16)	0.950
Treatment	43 (69.35)	39 (70.91)	1	131 (67.18)	80 (59.26)	0.162	-10 (-30, 10)	0.352
Controlling	19 (44.19)	19 (50)	0.659	49 (37.4)	51 (63.75)	< 0.001	26 (1, 51)	0.025

Additionally, we used the IPW Diff-in-Diff analysis to investigate the causal effect of FPP on diabetes mellitus as another type of NCDs. The analysis results to determine the causal effect of FPP on DM prevalence, screen, awareness, treatment, and control rates are shown in Table 4. Among the diabetes participants, FPP implementation had an increased causal effect on the DM screen (ATT = 20%, 95% CI: (6%, 34%)) and awareness (ATT = 14%, 95% CI: (1%, 27%)) compared to non-FPP. In addition, the FPP led to decrease in the treatment (ATT = -32%, 95% CI: (-59%, -5%)). However, DM prevalence and control in diabetes participants were constant.

**Table 4:** Effect of FPP on the prevalence, ever screening, awareness, treatment, and controlling of diabetes mellitus in the pre and post-policy periods

Outcome	Pre-policy (2011)			Post-policy (2016)			ATT (95% CI)	*P*-value
	non-FPP (*n* = 277)	FPP (*n* = 265)	*P*-value	non-FPP (*n* = 953)	FPP (*n* = 561)	*P*-value		
Prevalence	32 (11.55)	43 (16.23)	0.135	130 (13.64)	103 (18.36)	0.015	0 (-8, 7)	0.951
Ever screening	31 (96.88)	35 (81.4)	0.069	122 (94.57)	99 (97.06)	0.519	20 (6, 34)	0.004
Awareness	30 (96.77)	31 (88.57)	0.36	112 (91.8)	94 (94.95)	0.427	14 (1, 27)	0.042
Treatment	19 (63.33)	23 (74.19)	0.416	71 (66.36)	35 (42.17)	0.001	-32 (-59, -5)	0.012
Controlling	2 (20)	5 (25)	1	16 (32)	10 (45.45)	0.298	5 (-35, 44)	0.797

**Fig. 1:**
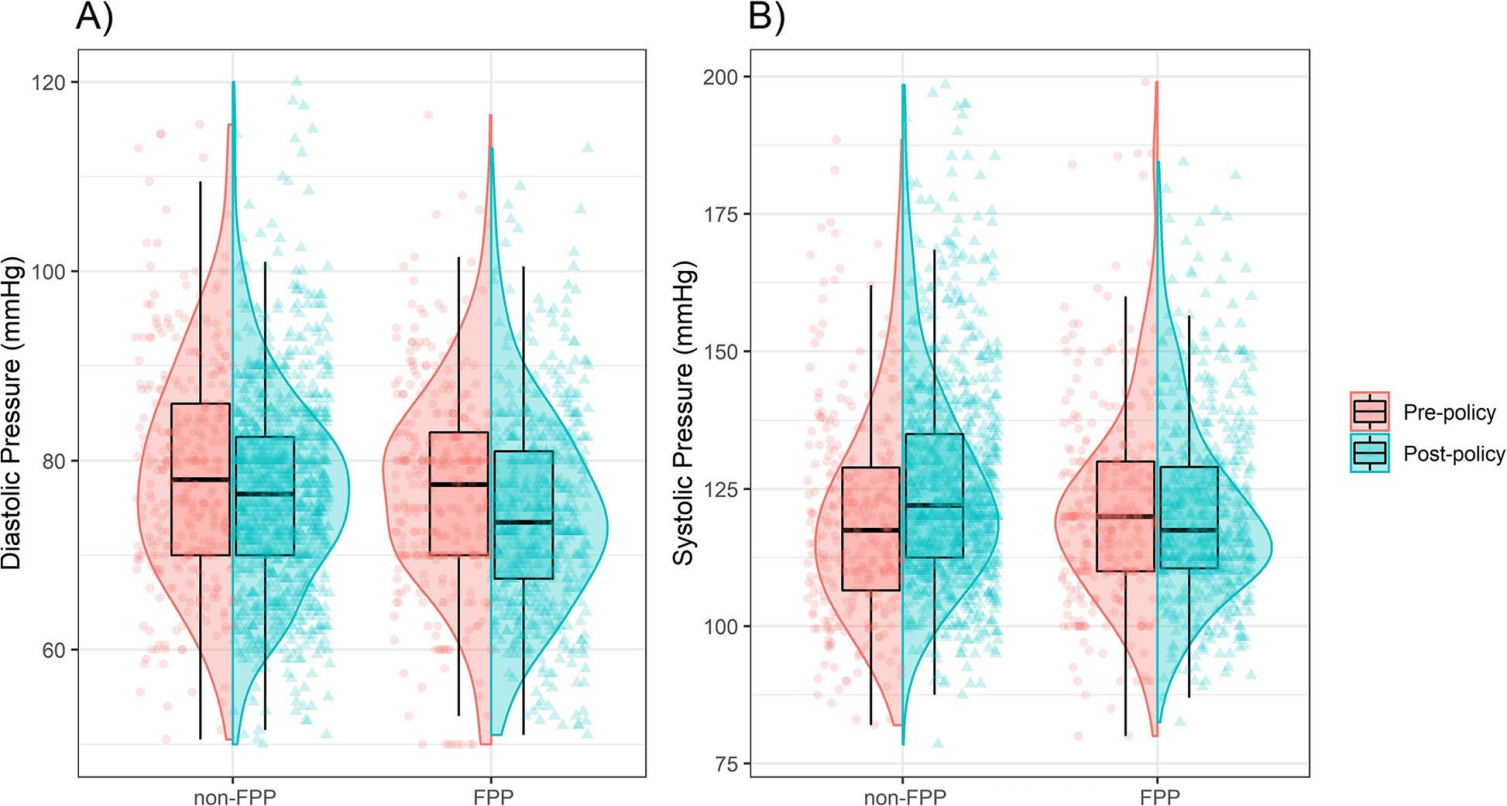
The distributions of systolic and diastolic in 2011 and 2016 in FPP and non-FPP administered cities

Figure 1 displays the distributions of systolic and diastolic blood pressure in 2011 and 2016 in FPP and non-FPP administered cities. The results indicate that in the FPP-administered city, the mean systolic blood pressure decreased from 121.47 mmHg in 2011 to 120.74 mmHg in 2016, while in the non-FPP city, the mean systolic blood pressure increased from 119.54 mmHg to 125.66 mmHg during the same period. These findings suggest that the FPP may have had a positive effect on controlling systolic blood pressure in the population. Similarly, the mean diastolic blood pressure decreased from 76.87 mmHg to 74.40 mmHg in the FPP city, whereas in the non-FPP city, the mean diastolic blood pressure decreased from 79.08 mmHg to 76.76 mmHg from the pre-policy period to the post-policy period. This implies that the FPP may have contributed to a better reduction in diastolic blood pressure levels in the population. Overall, the data show that participants living in the FPP-administered city had better blood pressure control compared to those living in non-FPP cities. The results of the TMLE analysis showed that FPP implementation had a decreased causal effect on the systolic (ATT = -6.75, 95% CI: (-8.66, -4.84)) and diastolic (ATT = -3.06, 95% CI: (-4.26, -1.85)) in whole sample (Fig. 1).

**Fig. 2:**
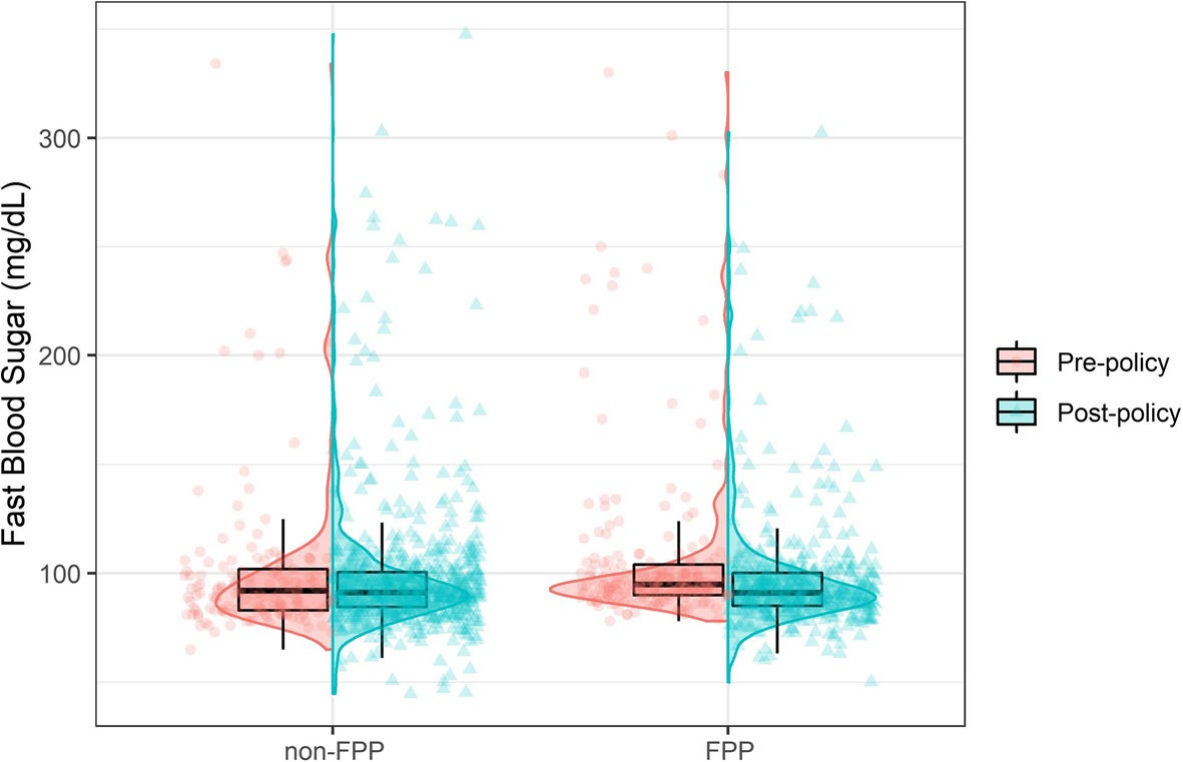
The distribution of Fast Blood Sugar (FBS) in 2011 and 2016 in FPP and non-FPP administered cities

Figure 2 illustrates the distribution of Fast Blood Sugar (FBS) levels in FPP and non-FPP administered cities in 2011 and 2016. The mean value of FBS decreased from 107.01 mg/dL to 97.35 mg/dL in the FPP administered city and from 101.64 mg/dL to 98.71 mg/dL in the non-FPP administered city. These findings suggest that participants residing in the FPP-administered city have better control over their FBS levels. However, the results of the TMLE analysis revealed that there was no significant change in FBS levels when the FPP was implemented compared to when it was not implemented. The average treatment effect on the treated (ATT) was -2.99, with a 95% confidence interval of (-6.72, 0.72). This indicates that the implementation of the FPP did not have a significant impact on FBS levels (ATT = -2.99, 95% CI: (-6.72,0.72)).

## Discussion

Hypertension and diabetes mellitus are major public health concerns in Iran, with high prevalence rates and a significant burden on the healthcare system. The FPP, which was executed in a part of Iran in 2012 as a pilot study, aims to improve access to primary healthcare services and prevent and control chronic diseases such as hypertension and diabetes mellitus. This study set out to investigate the causal effects of the FPP implementation on hypertension and diabetes mellitus among Iranian people. To do this, the study used two cross-sectional surveys taken from the STEPs survey before and after the FPP intervention in two cities, one of which was an FPP city and the other was a non-FPP city.

Generally, the results of this study demonstrate that FPP implementation in Iran has achieved some success in increasing the screening and control of HTN, as well as improving the screening and awareness of DM. However, the findings based on the JNC7 and 2017 ACC/AHA guidelines showed that this policy did not significantly improve hypertension prevalence, treatment, and control. On the other hand, there was a significant decrease in the average of SBP and DBP in the FPP-administrated city compared to non-FPP. Regarding diabetes mellitus, the nonsignificant ATT of DM prevalence and control over time and also the unexpected negative effect of FPP on the treatment of DM could show a weakness for the FPP, which may need to revise the policy guidelines.

This finding is consistent with some previous studies that have shown that FPPs, as a referral and monitoring system, can play an important role in improving the management of NCDs in Iran. Based on the study of Khadivi et al., FPP improved HTN and DM screening and case findings in rural areas of Isfahan, as a FPP-administered area of Iran [27]. However, this study did not use any causal methods to adjust for confounders, also did not use repeated surveys (before and after the policy) with a control group, and didn’t investigate different subgroups of HTN and DM.

The review study of Shirvani indicates that the Family Physician program in Iran has improved health indicators, increased healthcare access, reduced costs, and raised satisfaction rates. Yet, there are still deficiencies in the referral system, health record-keeping, diagnostic and therapeutic service registration, and public education that require attention and improvement [28].

Family physician policy has been implemented in several Asian countries. For example, Turkey, which shares a border with Iran and is located in Western Asia, launched a pilot program in 2003 to introduce a family medicine model. The program was later expanded to cover the entire country by 2010. According to research conducted by Sengul et al., this initiative significantly enhanced hypertension awareness, treatment, and control rates in Turkey from 2003 to 2012 [29–31]. Additionally, the Chinese government introduced the Health Care System Reform in the spring of 2009, which mandated that community health services must offer management services for chronic diseases. Huang et al. reported in 2019 that FPP had an indirect effect on Chinese patients with HTN and DM by increasing self-management behaviors [32]. Furthermore, Li et al. (2020) found that after implementing the family physician-optimized collaborative model in China, the mean systolic blood pressure decreased, and overall blood pressure control rate increased [33].

In some developed countries, such as Canada, the United States, the UK, and Germany, the FPP as a referral system is implemented [34–37]. Studies by Houlihan et al. and Tu declared that FPP improved the management of hypertension in Canada [38, 39]. In the United States, Foote et al. concluded that work-site hypertension programs in FPP can manage and improve the blood-pressure by including routine follow-up [36]. In Germany, the Munich Blood Pressure Program (MBP) was performed in 1983 to screen HTN patients and refer them to a family physician for subsequent management. Hense et al. reported that the MBP had an increase in the proportion of treated and controlled hypertensives [37].

As mentioned, in the present study the FPP could not have a sufficient effect in some aspects of the cascade of HTN and DM as NCDs. In the following, based on our results and existing literature, we will present some recommendations to enhance the effectiveness of FPP.

 The prevalence of HTN and DM can be influenced by FPs through health education and recommendations regarding the risk factors associated with HTN and DM. Some risk factors are unhealthy diet, overweight/obesity, excessive salt intake, insufficient physical activity, smoking, not getting enough sleep, and consumption of alcohol, tobacco, and other drugs [40–42].

The FPP in Iran has been able to improve the ever-screen of HTN, which, if accompanied by regular follow-up, could effectively control HTN and decrease the risk of complications associated with hypertension, as well as the burden of disease [43, 44].

Considering the evaluation, monitoring, and training system for health workers and FPs in order to adhere to guidelines is crucial, and it will have a positive effect on reaching the goal of FPP, such as increasing the awareness of patients with HTN. Increasing awareness of hypertension status can significantly accelerate the treatment initiation and thereby reduces the risk of complications associated with hypertension [43].

The nonsignificant effect of FPP on the HTN treatment cannot be considered a weak result for FPP, as it significantly increased HTN control. This may be due to non-pharmaceutical therapies, such as improving lifestyle and decreasing diet-related risk factors, such as eating a balanced diet, engaging in regular physical activity, and avoiding alcohol, tobacco, and other drugs. Furthermore, the lack of awareness regarding NCDs can result in delayed treatment, making it crucial for Family Physicians (FPs) to raise awareness, encourage lifestyle changes and promote regularity in taking pharmaceutical treatment [45, 46].

Despite the lack of clarity surrounding the advantages and disadvantages of implementing FPP and the incomplete evaluation of its effect on NCDs based on the pilot study, the government of Iran has decided to extend the FPP to all provinces in 2022 and 2023. While the specific factors that influenced this decision remain unclear, it is plausible that reduced health inequalities, health costs, and a desire to enhance public health outcomes were contributing factors. According to our results and previous studies that declared the limitations and challenges of FPP, this policy should be revised to improve the prevention, screening, awareness, treatment, and control of NCDs before expanding to other parts of Iran. The revision of the policy can be categorized into two general aspects.

The first aspect involvespromoting the performance of FPP. It includes increasing the number of primary health workers and medical equipment to allocate sufficient time for health-based care and follow-up the patients. In addition, training the health workers to improve the health education and lifestyle of participants, improving the satisfaction and motivation of the health workers and FPs by regular and sufficient payments, and an appropriate job environment. Moreover, increasing the referral system’s efficiency by redefining the feedback procedure between FPs and medical specialists, and managing the policy by monitoring and evaluating the performance of health workers.

The second aspect of the promotion is related to participants of FPP. It includes improving people's knowledge about the aims of FPPs, informing people about the benefits of this program to become interested in participation, and improving the authority and acceptability of FPs by increasing the mutual trust between FPs, medical specialists, and participants [15, 45–48].

The advantage of this study is that provides compelling evidence for policymakers to improve the guidelines and procedures for expanding the FP. This study's additional advantage lies in its utilization of an advanced double-robust statistical method to estimate the causal effect, as well as its combination of data from two STEPs surveys, which allowed for detailed information on confounders.

This study's limitation is that it only assessed the five-year impact of FPP on HTN and DM care cascade, and further studies with longer periods, using the updated version of STEPs, are required to fully evaluate the long-term effects of FPP. Notes that the absence of causal effect of FPP on DM control in this study may be due to the small sample size included in downstream of the cascade. Additionally, the present study was considered HTN and DM as two important non-communicable diseases. It seems that it is necessary to evaluate the effect of FPP on other type of NCDs. Finally, future research can explore the indirect impact of FPP on the management of HTN and DM, which could be useful in identifying the critical mediator factors.

## Conclusions

Hypertension and diabetes mellitus are two of the most prevalent non-communicable diseases worldwide, leading to a significant number of deaths each year. The implementation of the Family Physician Program in Iran aimed to provide individuals and families with comprehensive healthcare services. One of its primary objectives was to screen and raise awareness about hypertension and diabetes, which are essential for managing of these diseases.

Our study's findings indicate that the Family Physician Program in Iran has achieved some success in improving health outcomes. However, the program has some limitations and challenges, as evidenced by insufficient primary health workers and medical equipment, inadequate motivation and job satisfaction of health workers, cultural resistance of people to the integration of the referral system, and inadequate mutual trust between FPs, medical specialists, and participants, and etc. Therefore, it is essential to address these challenges, continuously evaluate and monitor the program, before expanding it to other regions of the country.

## Data Availability

The datasets used in the current study are not publicly available due to national rules and regulations but are available from the corresponding author (NR) on reasonable request.

## Abbreviations

2017 ACC/AHA: 2017 ACC/AHA/AAPA/ABC/ACPM/AGS/APhA/ASH/ASPC/NMA/PCNA Guideline for the Prevention, Detection, Evaluation, and Management of High Blood Pressure in Adults
ATT: Average treatment effect on treated
BMI: Body mass index
BP: Blood pressure
DBP: Diastolic blood pressure
Diff-in-Diff: Difference in Difference
DM: Diabetes mellitus
EMR: Eastern Mediterranean Region
FBS: Fast blood sugar
FPP: Family Physician Program
HTN: Hypertension
IPW: Inverse Probability Weighting
JNC7: The Seventh Joint National Committee on Prevention, Detection, Evaluation, and Treatment (JNC7) guidelines for the Management of High Blood Pressure in Adults
MET: Metabolic equivalents
NCD: Non-communicable diseases
PTA: Parallel trend assumption
SBP: Systolic blood pressure
SES: Socioeconomic status
STEPS: World Health Organization (WHO) STEPwise approach to Surveillance
TMLE: Targeted Maximum Likelihood Estimation
WHO: World Health Organization
WHR: Waist-to-Hip Ratio
